# A Population-Based Study to Evaluate the Associations of Nodal Stage, Lymph Node Ratio and Log Odds of Positive Lymph Nodes with Survival in Patients with Small Bowel Adenocarcinoma

**DOI:** 10.3390/curroncol29030110

**Published:** 2022-02-22

**Authors:** Atul Batra, Shiying Kong, Malek B. Hannouf, Winson Y. Cheung

**Affiliations:** 1 Department of Medical Oncology, Tom Baker Cancer Center, Calgary, AB T2N 4N2, Canada; batraatul85@aiims.edu; 2 Cumming School of Medicine, University of Calgary, Calgary, AB T2N 4N2, Canada; kristine621@outlook.com (S.K.); malek.hannouf@ucalgary.ca (M.B.H.)

**Keywords:** small bowel adenocarcinoma, small intestinal cancer, lymph node ratio, log odds of positive lymph nodes, discriminatory ability

## Abstract

Purpose: This study aimed to determine the real-world prognostic significance of lymph node ratio (LNR) and log odds of positive lymph nodes (LOPLN) in patients with non-metastatic small bowel adenocarcinoma. Methods: Patients diagnosed with early-stage small bowel adenocarcinoma between January 2007 and December 2018 from a large Canadian province were identified. We calculated the LNR by dividing positive over total lymph nodes examined and the LOPLN as log ([positive lymph nodes + 0.5]/[negative lymph nodes + 0.5]). The LNR and LOPLN were categorized at cut-offs of 0.4 and −1.1, respectively. Multivariable Cox proportional hazards models were constructed for each nodal stage, LNR and LOPLN, adjusting for measured confounding factors. Harrell’s C-index and Akaike’s Information Criterion (AIC) were used to calculate the prognostic discriminatory abilities of the different models. Results: We identified 141 patients. The median age was 67 years and 54.6% were men. The 5-year overall survival rates for patients with stage I, II and III small bowel adenocarcinoma were 50.0%, 56.6% and 47.5%, respectively. The discriminatory ability was generally comparable for LOPLN, LNR and nodal stage in the prognostication of all patients. However, LOPLN had higher discriminatory ability among patients with at least one lymph node involvement (Harrell’s C-index, 0.75, 0.77 and 0.82, and AIC, 122.91, 119.68 and 110.69 for nodal stage, LNR and LOPLN, respectively). Conclusion: The LOPLN may provide better prognostic information when compared to LNR and nodal stage in specific patients.

## 1. Introduction

Small intestinal cancers comprise 0.6% of all new cancer diagnoses and 0.3% of all cancer deaths in the United States. Approximately 11,000 new diagnoses and 1700 deaths are expected in 2020 [1]. Adenocarcinoma and neuroendocrine tumours represent the two most common histologies, each accounting for approximately 40% of small intestinal cancers, with the remainder largely consisting of lymphoma and sarcoma [2,3,4]. Further, small bowel adenocarcinoma contributes to less than 5% of all cancers of the digestive tract [5]. Of these, the most frequent primary site is the duodenum (60%) followed by the jejunum (25%) and the ileum (15%) [6,7].

The prognosis of small bowel adenocarcinoma continues to be poor, with reported 5-year overall survival rates ranging from 14 to 33% [4,6,7,8]. Complete resection of the primary tumour along with regional lymph node dissection remains the only curative treatment [2]. Lymph node invasion has been reported as the most significant prognostic factor [6,7]. Similar to the colorectal cancer setting, the number of lymph nodes that are retrieved and examined in the surgical specimen is also considered to offer prognostic value [9,10,11]. While the optimal extent of lymph node dissection in small bowel adenocarcinoma is unclear, a recently published study suggested that resection of at least 12 lymph nodes, akin to recommendations used for large bowel cancer, is likely to improve the survival in patients with jejunal and ileal adenocarcinomas [10].

The American Joint Committee on Cancer (AJCC) nodal staging of digestive tract tumours is based on the number of positive lymph nodes and does not account for the number of lymph nodes examined [12]. For instance, the eighth edition of the AJCC categorizes the nodal stage in small bowel adenocarcinoma as N0, N1 and N2 to reflect 0, 1 to 2, and more than 2 positive lymph nodes, respectively. In an effort to improve prognostication further, researchers have attempted to evaluate the value of other scores, such as the proportion of positive to total lymph nodes resected, which is known as the lymph node ratio (LNR) [11,13,14]. The LNR has been validated as a prognostic marker in digestive tract malignancies including colorectal, gastric, oesophageal and pancreatic cancer [13,14,15,16,17], but the uptake of LNR in staging has been variable.

One reason is that the utility of LNR is limited to patients with at least one positive lymph node because the LNR is 0 irrespective of the number of lymph nodes resected among patients without nodal involvement. Thus, the log odds of positive lymph nodes (LOPLN) was developed. This is calculated as the log of (positive lymph nodes + 0.5/negative lymph nodes + 0.5) [13,18,19]. The published literature on patients with colorectal cancer has suggested that LOPLN provides better prognostic discriminative ability when compared with LNR and AJCC nodal stage [13,18,19].

Therefore, this study aimed to assess the associations of lymph node involvement and extent of lymph node resection with survival in patients with small bowel adenocarcinoma. Specifically, we compared the prognostic discriminative ability of AJCC nodal staging, LNR, and LOPLN as measures of lymph node burden.

## 2. Methods

### 2.1. Study Design and Data Sources

This was a retrospective, population-based study conducted in Alberta, Canada. Alberta is a large Canadian province with over four million residents. The Alberta Cancer Registry is responsible for the prospective collection of data regarding patients diagnosed with cancer. The data consist of demographic variables, tumour characteristics, treatment patterns, and survival outcomes.

### 2.2. Study Population

Patients diagnosed with non-metastatic small bowel adenocarcinoma in Alberta, Canada from January 2007 to December 2018 were included in the current study. Patients who emigrated from the province and those with multiple primary malignancies were excluded. The results of this study are reported as per the STROBE (Strengthening the Reporting of Observational Studies in Epidemiology) guidelines [20]. The study was approved by the Health Research Ethics Board of Alberta’s Cancer Committee.

### 2.3. Clinical Variables and Outcomes

Baseline demographic variables including age, sex, and one of five predefined health zones were examined. Tumour characteristics were summarized based on the site of the primary (duodenum, jejunum or ileum), the histopathological subtype, the grade (low, I/II vs. high, III/IV), the presence of lymphovascular invasion, the depth of invasion of the primary tumour, the number of lymph nodes resected and the number of lymph nodes positive for tumour involvement. Further, we grouped the number of resected lymph nodes into none, 0 to 11, and at least 12, based on similar classifications used in large bowel tumours [21,22,23]. Data on the local invasion of the primary tumour and the number of positive lymph nodes were used to categorize patients according to the eighth edition of the AJCC tumour stage (T1 to T4), nodal stage (N0 to N2) and overall stage groups (I to III) [12].

Lymph node burden was characterized using three different metrics. First, we used the AJCC nodal staging system and classified patients as having N0, N1 or N2 disease. Second, LNR was calculated by dividing the number of lymph nodes showing tumour deposits by the number of resected lymph nodes [14]. While various cut-offs have been used to categorize LNR, we used a single cut-off because of our sample size. The cut-off of 0.4 was selected based on two large studies in patients with colon cancer [24,25]. Finally, LOPLN was calculated by log ([number of positive lymph nodes + 0.5]/[number of negative lymph nodes + 0.5]) [26]. The number of negative lymph nodes was calculated by subtracting positive nodes from the total number of resected nodes. A value of 0.5 was added to both the numerator and the denominator as recommended in previous publications to avoid dividing by 0 and to reduce the number of patients with a LOPLN of 0 [13,26]. Similar to LNR, there are multiple potential cut-offs to categorize LOPLN in colon cancer [18,27]. We used a single cut-off of −1.1, which was based on the cut-off used in a previous study that was nearest to the median value in our cohort [19].

Endpoint included overall survival, which was defined as the interval from the date of diagnosis to the date of death due to any cause, censoring at last known follow-up.

### 2.4. Statistical Analysis

Baseline characteristics including demographic and tumour related variables were analysed using descriptive statistics. Patients who did not have any lymph nodes resected were excluded from the survival analysis. Kaplan–Meier curves were used to determine overall survival and log rank tests were conducted to analyse differences across categories of AJCC nodal stage, LNR and LOPLN. We also performed survival analysis in a subpopulation of patients with at least one positive lymph node affected by cancer. Cox proportional hazard models were constructed to analyse the effect of nodal burden on survival outcomes, while adjusting for measured confounders. We also developed separate Cox models for AJCC nodal stage, LNR and LOPLN in all patients and in those with involved lymph nodes only. We calculated the Harrell’s Consistency Index (C-index) and the Akaike’s Information Criterion (AIC) to assess the relative discriminative abilities of AJCC nodal stage, LNR and LOPLN. A higher Harrell’s C-index indicates a better discriminative ability whereby a value of 0.5 indicates no discriminatory power and a value of 1 indicates complete differentiation [28]. Further, AIC assigns a relative value to each model. No cut-offs exist to distinguish a good vs. poor model, but a lower value represents a better overall fit of the model [29]. All statistical tests performed in this study were two-sided and the significance level was defined a priori as <0.05. All analyses were performed using Stata statistical software (StataCorp. 2013. Release 13. College Station, TX, USA).

## 3. Results

### 3.1. Patient Characteristics

We identified 141 patients with non-metastatic small bowel cancer who were diagnosed and treated in Alberta from January 2007 to December 2018. The median age at diagnosis was 67 years (interquartile range, 30–89 years) and 64 (45.4%) patients were women. The most common primary site was the duodenum in 78 (55.3%) patients, followed by the ileum and jejunum in 37 (26.2%) and 26 (18.4%) patients, respectively.

Of all the patients, 116 (82.3%) were treated with a resection of the primary tumour accompanied by dissection of the regional lymph nodes. In contrast, 23 (16.3%) patients underwent surgery of the small bowel cancer only and two (1.4%) patients had lymph node aspiration along with the removal of the primary tumour. A low histological grade was noted in 88 (76.5%) patients and a high grade was reported in 27 (23.5%) cases. Lymphovascular invasion was present in 42 (48.3%) patients. The median number of resected lymph nodes was 15 (interquartile range, 2–38) and 61.2% had more than 12 nodes removed.

Complete staging information was available in 115 patients, of which 71 (61.7%) had stage III small bowel cancer while 6 (5.2%) and 38 (33.0%) had AJCC stage I and II disease, respectively. In terms of tumour staging, 107 (93.0%) patients had T3/T4 disease, while T1/T2 stages were reported in eight (7.0%) patients (Table 1).

### 3.2. Lymph Node Burden

With respect to nodal staging, there were 45 (38.8%), 23 (19.8%) and 48 (41.4%) patients with N0, N1 and N2 disease, respectively. Twenty-eight (50.0%), eight (32.0%) and twelve (34.3%) patients with duodenal, jejunal and ileal adenocarcinomas had N2 stage, respectively.

The scores were categorized as ≤0.4 and >0.4 for LNR and ≤−1.1 and >−1.1 for LOPLN based on the cut-offs suggested in previous publications, and for LOPLN, closest to the median value of our cohort. Around three-fourths of all patients had LNR ≤0.4 and 59.5% had LOPLN <−1.1. Further, 67.8%, 76.0% and 80.0% of patients with duodenal, jejunal and ileal adenocarcinomas had LNR ≤0.4. The corresponding percentages for LODDS <−1.1 were 48.2%, 76.0% and 65.7%, respectively (Table 2).

### 3.3. Survival Outcomes in Patients Who Underwent Lymph Node Dissection

We limited the survival analysis to the 116 patients who underwent small bowel resection with lymph node dissection. At a median follow-up of 64 months, 52 deaths were reported due to all causes. The 5-year overall survival rate of patients who underwent complete surgery was 51.3%. By AJCC stage, the 5-year overall survival rate was 50.0%, 56.6% and 47.5% for stages I, II and III (*p* = 0.571), respectively.

**Figure 1:**
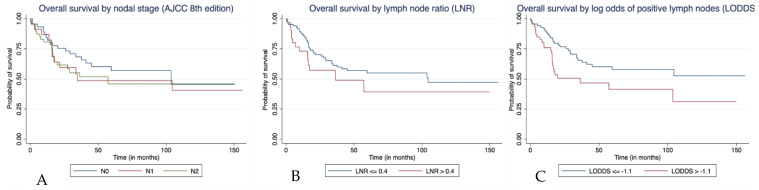
Kaplan–Meier curves showing overall survival of patients who underwent resection of primary small bowel adenocarcinoma with at least one lymph node sampled in the pathological specimen (n = 116) by (**A**) AJCC nodal stage (**B**) LNR, and (**C**) LOPLN.

We constructed three separate models for overall survival using AJCC nodal stage, LNR and LOPLN, respectively. All other baseline and tumour characteristics were kept constant as confounding variables in each of the models. Primary jejunal tumours had significant better overall survival in all three models (*p* = 0.004, *p* = 0.014 and *p* = 0.005, respectively). A higher nodal burden as measured by AJCC nodal stage (N2; hazard ratio [HR], 3.71; 95% confidence interval [CI], 1.19–11.57; *p* = 0.024), LNR (HR, 4.39; 95% CI, 1.33–14.50; *p* = 0.015) and LOPLN (HR, 5.97; 95% CI, 1.92–18.57; *p* = 0.002) (Figure 1A–C) all predicted worse overall survival. There were no associations among other variables, including age, sex, AJCC tumour stage, grade, lymphovascular invasion, or number of lymph nodes resected, with overall survival (Table 3).

Further, we compared the discriminatory value of the three models. The Harrell’s C indices were 0.75, 0.75 and 0.76 for models with AJCC nodal stage, LNR and LOPLN, respectively. Likewise, the AIC values were 241.86, 239.95 and 236.27 for the corresponding models, respectively.

### 3.4. Factors Predicting Survival in Patients with Positive Lymph Nodes

**Figure 2:**
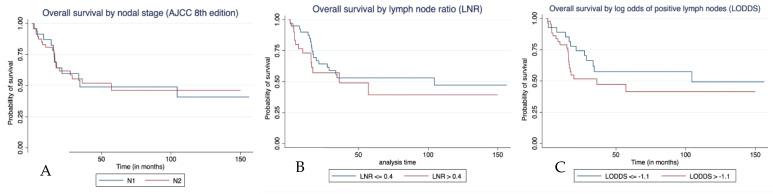
Kaplan–Meier curves showing overall survival of patients who underwent resection of primary small bowel adenocarcinoma with at least one lymph node affected by tumour (n = 71) by (**A**) AJCC nodal stage (**B**) LNR, and (**C**) LOPLN.

We constructed multivariable Cox proportional hazards models to examine the associations of AJCC nodal stage, LNR and LOPLN with overall survival in the subgroup of 71 patients who had at least one lymph node involved by small bowel cancer. Of note, nodal burden by AJCC stage (N2 vs. N1; HR, 1.82; 95% CI, 0.56–5.93; *p* = 0.317) did not predict overall survival, while LNR > 0.4 (HR, 5.64; 95% CI, 1.02–30.90; *p* = 0.046) and LOPLN > −1.1 (HR, 31.75; 95% CI, 4.39–234.16; *p* = 0.001) predicted inferior overall survival in the respective models (Figure 2A–C). Primary jejunal tumours had better outcomes in all three models (*p* = 0.008, *p* = 0.021 and *p* = 0.003). In the Cox model with LOPLN, male sex (HR, 8.54; 95% CI, 1.40–54.63; *p* = 0.020) and T4 stage (HR, 8.80; 95% CI, 2.26–34.32; *p* = 0.002) predicted worse overall survival (Table 4). The Harrell’s C indices were 0.75, 0.77 and 0.82 for AJCC nodal stage, LNR and LOPLN, respectively. The AIC values were 122.91, 119.68 and 110.69, respectively.

### 3.5. Factors Predicting Survival in Patients without Lymph Node Involvement

There were 45 patients without lymph node involvement by tumour. Older age at diagnosis was associated with worse overall survival (HR, 1.08; 95% CI, 1.00–1.17; *p* = 0.041). However, no other baseline factors including sex, primary tumour site, AJCC tumour stage, grade, lymphovascular invasion and number of lymph nodes resected was associated with overall survival. Of note, the median number of lymph nodes removed in this subgroup of patients was 12 (interquartile range, 2–23) and 53.3% patients had at least 12 lymph nodes resected (Table S1).

### 3.6. Survival Outcomes of Patients without Lymph Node Dissection

There were 25 patients treated with resection of primary tumour without any lymph nodal dissection. Of these, 19 patients died of cancer and two patients succumbed due to non-cancer causes. The median overall survival of this group of patients was 11.9 months (95% CI, 7.4–18.8 months).

## 4. Discussion

In this study, patients who were treated without lymph node dissection had dismal outcomes with median overall survival of less than one year. Patients with primary jejunal adenocarcinoma had better survival outcomes compared with duodenal and ileal tumours. While the AJCC nodal stage N2 was associated with worse survival compared to N0, there was no difference in survival based on nodal stage (N1/N2) among patients with positive lymph nodes. In contrast, a higher LNR (>0.4) and LOPLN (>−1.1) predicted worse survival in patients with lymph nodal involvement and in the overall population. The discriminative ability and overall fit for overall survival were better for LOPLN when compared to LNR and the AJCC nodal stage. Of note, age was the only characteristic associated with overall survival in patients without lymph node involvement.

The 5-year overall survival has been previously reported as 77.7%, 43.7% and 24.9% for patients with stage I, II and III small bowel adenocarcinomas, respectively [6]. In comparison, our patients had a lower 5-year overall survival rate for patients with stage I small bowel adenocarcinoma, but higher for those with stage II and III disease. This is likely because there were only six patients with stage I disease in our study and there were three deaths, of which two were cancer related. Of note, the median number of lymph nodes examined in our patients was 15 and over 60% of patients had more than 12 nodes resected with the primary tumour. The extent of resection of lymph nodes is one of the most significant prognostic factors, which may explain the higher 5-year overall survival rates seen in our patients [10,11].

Although all three measures of lymph nodal involvement by tumour were associated with overall survival of patients with small bowel adenocarcinoma, the discriminatory ability of LOPLN was higher than the AJCC nodal stage and LNR, especially in patients with lymph node-positive tumours. Further, the AJCC nodal stage (N1 vs. N2) failed to predict survival when only node-positive patients were analysed. This highlights the fact that the AJCC nodal stage considers only the positive lymph nodes and therefore patients with 2/2 positive lymph nodes and 2/15 positive lymph nodes will be staged similarly as N1 [12]. However, LNR and LOPLN consider both positive lymph nodes and number of resected lymph nodes and therefore, have a higher discriminatory ability [14,18]. The higher discriminatory value of LOPLN over LNR and the AJCC nodal stage has been consistently demonstrated in previous studies conducted in patients with colorectal cancer [13,26,30]. A prior study also demonstrated the prognostic value of LOPLN over LNR and nodal stage in patients with small bowel adenocarcinoma diagnosed from 1988 to 2010 [31]. In comparison, our study represents a more contemporary set of patients (2007 to 2018). Further, the median number of lymph nodes retrieved in the prior study was 8 compared to 15 in our patients. This likely represents acknowledgement of the prognostic significance of number of lymph nodes resected in patients with small bowel adenocarcinoma over the last decade.

Patients with jejunal cancer had better overall survival compared to those with primary duodenal cancer. Previous studies have reported that patients with duodenal adenocarcinoma have worse prognosis compared to those with jejunal and ileal tumours [7,11,32]. However, we did not find any association of ileal primary tumours compared to duodenal adenocarcinoma.

Lastly, we did not find any prognostic significance of number of lymph nodes resected in patients without lymph nodal involvement. While this has been reported as a prognostic marker in previous studies, this was a small subset in our study [10,11]. Moreover, the median number of resected lymph nodes was 12 and 53.3% of patients in this subset had at least 12 lymph nodes resected with the primary tumour, so the propensity for patients in our cohort to have undergone aggressive lymph node dissection may have tempered our ability to observe any differences.

We suggest a total of at least 10 lymph nodes be resected in clinically N0/N1 disease, as this will provide a LOPLN value of less than −1.1 with a maximum of two positive lymph nodes. In patients with clinical N2 disease, resection of 21 nodes with five involved nodes gives a borderline value of −1.1. However, the actual number of involved lymph nodes is difficult to predict preoperatively.

The study was limited by its retrospective design and the use of administrative data sources. The sample size, although small in absolute number, represents one of the largest real-world studies to date because of the rarity of small bowel adenocarcinoma. Further, the median age of our patients was 67 years, and therefore, many patients would likely be affected by at least one comorbid condition. However, data on performance status and comorbid conditions were not available, which can independently affect the survival outcomes. The details on the type of surgery for different subsites were not available and it is possible that a more extensive lymph node dissection was preferred for some tumours. Lastly, we used single cut-off of LOPLN, which was based on prior publications and was close to the median values of our patients. This limits the generalizability of the cut-off to external cohorts and would need further validation.

## 5. Conclusions

In conclusion, lymph node dissection is of paramount importance while treating small bowel adenocarcinoma. Use of indices that consider the number of lymph nodes resected in combination with the number of positive lymph nodes appears to provide better prognostic discriminatory ability. Routine use of LOPLN, especially in patients with positive lymph nodes, should be supported.

## Figures and Tables

**Table 1:** Baseline characteristics of patients with non-metastatic small bowel cancer (n = 141).

Variable	N (%)
Age at diagnosis, in years	
Median	67
Interquartile range	30–89
Sex	
Females	64 (45.4%)
Males	77 (54.6%)
Site	
Duodenum	78 (55.3%)
Jejunum	37 (26.2%)
Ileum	26 (18.4%)
Histopathological subtype	
Adenocarcinoma	103 (73.0%)
Adenocarcinoma in tubulovillous adenoma	11 (7.8%)
Adenocarcinoma in adenomatous polyp	9 (6.4%)
Adenocarcinoma in villous adenoma	4 (2.8%)
Mucinous	9 (6.4%)
Intestinal	3 (2.1%)
Adenocarcinoma with mixed subtypes	2(1.4%)
Lymph nodes resected	
0	25 (17.7%) *
1–11	45 (31.9%)
≥12	71 (50.4%)
AJCC (8th edition) Stage (n = 115)	
I	6 (5.2%)
II	38 (33.0%)
III	71 (61.7%)
AJCC (8th edition) T stage (n = 115)	
T1	4 (3.5%)
T2	4 (3.5%)
T3	49 (42.6%)
T4	58 (50.4%)
Histological grade (n = 115)	
Grade I/II	88 (76.5%)
Grade III/IV	27 (23.5%)
Lymphovascular invasion (n = 87)	
Yes	42 (48.3%)
No	45 (51.7%)
Zone of residence	
Calgary	51
Central	39
Edmonton	21
North	19
South	6
Unknown	5

* 23 patients were treated without lymph node resection and two were treated with aspiration from lymph nodes.

**Table 2:** Lymph nodes positivity by different methods, n = 116.

Variable	Parameter	Duodenal	Jejunal	Ileal
		(n = 56)	(n = 35)	(n = 25)
AJCC N stage (8th edition)				
N0	45 (38.8%)	15 (26.8%)	16 (45.7%)	14 (56.0%)
N1	23 (19.8%)	13 (23.2%)	7 (20.0%)	3 (12.0%)
N2	48 (41.4%)	28 (50.0%)	12 (34.3%)	8 (32.0%)
Lymph Node ratio (LNR)				
<0.4	85 (73.3%)	38 (67.9%)	28 (80.0%)	19 (76.0%)
>0.4	31 (26.7%)	18 (32.1%)	7 (20.0%)	6 (24.0%)
Log odds of positive lymph nodes				
(LOPLN)				
<−1.1	69 (59.5%)	27 (48.2%)	23 (65.7%)	19 (76.0%)
>−1.1	47 (40.5%)	29 (51.8%)	12 (34.3%)	6 (24.0%)

**Table 3:** Factors associated with overall survival in all patients (n = 116).

Variable	HR (95% CI)	*p*-Value	HR (95% CI)	*p*-Value	HR (95% CI)	*p*-Value
Age at diagnosis	1.03 (0.99–1.06)	0.111	1.02 (0.99–1.06)	0.123	1.02 (0.99–1.05)	0.129
Sex						
Female						
Male	1.33 (0.56–3.15)	0.511	1.21 (0.52–2.84)	0.655	1.68 (0.7–4.07)	0.246
Primary site						
Duodenum						
Jejunum	0.16 (0.04–0.56)	0.004	0.20 (0.06–0.72)	0.014	0.15 (0.04–0.56)	0.005
Ileum	1.73 (0.69–4.36)	0.242	1.49 (0.60–3.74)	0.390	1.70 (0.66–4.38)	0.273
AJCC tumour stage						
T1						
T2	0.74 (0.04–14.93)	0.842	0.88 (0.04–17.58)	0.933	1.08 (0.05–21.98)	0.96
T3	0.64 (0.07–5.69)	0.689	0.75 (0.09–6.43)	0.795	0.63 (0.07–5.50)	0.677
T4	1.24 (0.14–11.06)	0.845	1.83 (0.22–15.44)	0.577	1.65 (0.19–13.94)	0.647
Grade						
1–2						
3–4	0.78 (0.25–2.46)	0.673	0.68 (0.19–2.36)	0.541	0.42 (0.11–1.53)	0.187
Lymphovascular invasion						
No						
Yes	0.97 (0.36–2.60)	0.952	1.05 (0.41–2.71)	0.917	1.19 (0.47–3.01)	0.707
Lymph nodes resected						
<12						
≥12	0.86 (0.37–1.99)	0.724	1.50 (0.59–3.85)	0.392	1.61 (0.66–3.92)	0.299
AJCC Nodal stage						
0						
1	1.83 (0.61–5.45)	0.280				
2	3.71 (1.19–11.57)	0.024				
Lymph node ratio (LNR) ≤0.4						
>0.4				4.39 (1.33–14.05)		
Log odds of positive lymph nodes (LOPLN)						
≤−1.1					5.97 (1.92–18.57)	0.002
>−1.1						

HR: hazard ratio; CI; confidence interval.

**Table 4:** Factors associated with overall survival in node-positive patients (n = 71).

Variable	HR (95% CI)	*p*-Value	HR (95% CI)	*p*-Value	HR (95% CI)	*p*-Value
Age at diagnosis	0.99 (0.95–1.04)	0.833	1.01 (0.96–1.07)	0.732	1.03 (0.97–1.09)	0.276
Sex						
Female						
Male	1.22 (0.32–4.62)	0.765	1.72 (0.36–8.34)	0.500	8.54 (1.40–54.63)	0.020
Primary site						
Duodenum						
Jejunum	0.06 (0.01–0.47)	0.008	0.08 (0.01–0.69)	0.021	0.03 (0.03–0.30)	0.003
Ileum	1.15 (0.27–4.90)	0.850	1.13 (0.24–5.39)	0.879	2.99 (0.63–14.24)	0.170
AJCC T stage						
T1/2	-					
T3	Ref		Ref		Ref	
T4	2.44 (0.73–8.11)	0.146	3.81 (1.13–12.87)	0.031	8.80 (2.26–34.32)	0.002
Grade						
1–2						
3–4	0.78 (0.22–2.81)	0.707	0.41 (0.07–2.39)	0.320	0.06 (0.01–0.56)	0.014
Lymphovascular invasion						
No						
Yes	1.00 (0.23–4.25)	0.997	0.75 (0.17–3.38)	0.709	1.11 (0.30–4.09)	0.876
Lymph nodes resected						
<12						
≥12	0.86 (0.25–3.00)	0.815	2.28 (0.43–12.07)	0.333	4.48 (0.86–23.50)	0.076
AJCC Nodal stage						
1	Ref					
2	1.82 (0.56–5.93)	0.317				
Lymph node ratio (LNR) ≤0.4						
>0.4			5.64 (1.02–30.90)	0.046		
Log odds of positive lymph nodes (LOPLN)						
≤−1.1					31.75 (4.30–234.16)	0.001
>−1.1						

HR: hazard ratio; CI; confidence interval.

## Data Availability

The data presented in this study are available on request from the corresponding author. The data are not publicly available due to privacy concerns.

## Supplementary Materials

The following supporting information can be downloaded at https://www.mdpi.com/article/10.3390/curroncol29030110/s1: Table S1: Factors associated with overall survival in lymph nodes negative patients (n = 45).

## Abbreviations

AJCC	American Joint Committee on Cancer
LNR	Lymph Node Ratio
LOPLN	Log Odds Of Positive Lymph Nodes
STROBE	Strengthening the Reporting of Observational Studies in Epidemiology
AIC	Akaike’s Information Criterion
HR	Hazard Ratio
CI	Confidence Interval
