# Supplementary material for: A Population-Based Study to Evaluate the Associations of Nodal Stage, Lymph Node Ratio and Log Odds of Positive Lymph Nodes with Survival in Patients with Small Bowel Adenocarcinoma
Source: Curr Oncol. 2022 Feb 22;29(3):1298–308. doi: 10.3390/curroncol29030110 (PMC8947592; doi:10.3390/curroncol29030110)
Supplement: Supplementary file 1 [file curroncol-29-00110-s001.zip › curroncol-1585920-supplementary.pdf]

Current Oncology

Supplementary Materials

A Population-Based Study to Evaluate the Associations of Nodal Stage, Lymph Node Ratio and Log Odds of Positive Lymph Nodes with Survival in Patients with Small Bowel Adenocarcinoma

Atul Batra, Shiying Kong, Malek B. Haneef and Winson Y. Cheung

Table S1. Factors associated with overall survival in lymph nodes negative patients (n=45).

Variable	Overall survival	
	Hazard ratio (95% confidence interval)	P-value
**Age at diagnosis**	1.08 (1.00-1.17)	0.041
Sex		
Female		
Male	1.29 (0.35-4.80)	0.703
**Primary site**		
Duodenum		
Ileum	3.91 (0.67-22.96)	0.131
Jejunum	0.98 (0.14-6.90)	0.985
**AJCC T stage**		
T1/2		
T3	0.31 (0.03-3.10)	0.319
T4	0.45 (0.03-5.81)	0.541
**Grade**		
1-2		
3-4	0.23 (0.01-4.62)	0.336
**Lymphovascular invasion**		
No		
Yes	1.49 (0.35-6.39)	0.589
**Lymph nodes resected**		
<12		
≥12	0.50 (0.11-2.31)	0.372
